# *De novo* genome assembly of the tobacco hornworm moth (*Manduca sexta)*

**DOI:** 10.1093/g3journal/jkaa047

**Published:** 2021-01-28

**Authors:** Ariel Gershman, Tatiana G Romer, Yunfan Fan, Roham Razaghi, Wendy A Smith, Winston Timp

**Affiliations:** 1 Department of Molecular Biology and Genetics, Johns Hopkins University, Baltimore, MD, 21287, USA; 2 Department of Biomedical Engineering, Johns Hopkins University, Baltimore, MD, 21218, USA; 3 Department of Biology, Northeastern University, Boston, MA, 02215, USA

**Keywords:** *Manduca sexta*, Sphingid moth, genome, sequencing, assembly

## Abstract

The tobacco hornworm, *Manduca sexta*, is a lepidopteran insect that is used extensively as a model system for studying insect biology, development, neuroscience, and immunity. However, current studies rely on the highly fragmented reference genome Msex_1.0, which was created using now-outdated technologies and is hindered by a variety of deficiencies and inaccuracies. We present a new reference genome for *M. sexta*, JHU_Msex_v1.0, applying a combination of modern technologies in a *de novo* assembly to increase continuity, accuracy, and completeness. The assembly is 470 Mb and is ∼20× more continuous than the original assembly, with scaffold N50 > 14 Mb. We annotated the assembly by lifting over existing annotations and supplementing with additional supporting RNA-based data for a total of 25,256 genes. The new reference assembly is accessible in annotated form for public use. We demonstrate that improved continuity of the *M. sexta* genome improves resequencing studies and benefits future research on *M. sexta* as a model organism.

## Introduction

As a large, easily grown insect, the tobacco hornworm (*M. sexta, NCBI: txid7130)* is a key model for studying biochemical mechanisms in insect biochemistry, physiology, neurobiology, development, and immunity (Kay *et al.* 1992; Truman and Reiss 1995; Wieczorek *et al.* 1999; Mechaber *et al.* 2002). In particular, *M. sexta is* a well characterized model system for studying programmed cell death and metamorphosis (Jones *et al.* 1995). For example, the term “autophagic cell death” was introduced while studying metamorphic degeneration of muscle tissue in silkmoths (Lockshin and Williams 1965). Since then, the autophagic pathway has been studied extensively during the metamorphosis of many other lepidopterans. The size of *M. sexta* makes it an optimal system for studying the biochemical pathways associated with programmed cell death, but the system is limited by the quality of the reference genome. *M. sexta’*s innate immune pathways, many of which share mammalian homologs, have been characterized in detail and used as a model system for studying fungal virulence and drug efficacy (Kanost *et al.* 2004; Lyons *et al.* 2020). Finally, *M. sexta* is useful for understanding serine proteases, a repertoire of proteins involved in mediating defence responses such as hemolymph clotting, melanotic encapsulation, food digestion, antimicrobial peptide induction, and cytokine activation (Jiang *et al.* 2010; Cao *et al.* 2015). The serine protease gene family constitutes a large protein family in insects containing 50–300 genes (Cao *et al.* 2015). In addition to basic biology, more study of this insect has practical applications; *M. sexta* is a prevalent agricultural pest that feeds on solanaceous plants, *e.g.*, tobacco, and has the unique ability to tolerate large amounts of solanaceous alkaloids, such as nicotine (de Boer and Hanson 1987). Recent advancements in genomic sequencing have led to chromosome level reference genomes of other lepidopterans such as the domestic silkworm (*Bombyx mori*) and the monarch butterfly (*Danaus plexippus*) (Gu *et al.* 2019; Kawamoto *et al.* 2019*).*However, despite the importance of *M. sexta* as a model organism, no improvements to its reference assembly have been made since its release in 2016.

The current *M. sexta* genome is relatively fragmented, due to the abundance of repetitive sequences contained in most eukaryotic genomes. This lessens the value of the reference due to incomplete gene sequences and unanchored or mispositioned contigs on chromosomes. The first effort to sequence and assemble the *M. sexta* genome resulted in the Msex_1.0 assembly (GCA_000262585.1), a 419.4 Mb genome composed of 20,869 scaffolds with the largest scaffold being just 3.2 Mb (N50 = 664 Kb). The accompanying gene set comprised of 15,451 protein-coding genes, of which 2498 were manually curated (Kanost *et al.* 2016). This level of fragmentation in the assembly can lead to erroneous gene annotations and mis-mapping of resequencing data, therefore, slowing progress in Lepidopteran genomics (Denton *et al.* 2014). The continuity of the *M. sexta* reference assembly significantly lags behind other lepidopteran reference genomes such as the domestic silkworm (*B. Mori)*, a 460 Mb genome with a scaffold N50 of 16.8 Mb (Kawamoto *et al.* 2019). Because *M. sexta* is a model species in lepidoptera, its genome assembly needs to be highly accurate and contiguous to allow for comparative genomics studies of insect diversity, biochemical studies on homologous mammalian pathways, and agricultural studies regarding the tomato and tobacco crops, a food source for *M. sexta.*

Utilizing advancements in sequencing technology, we generated a new chromosome level *M. sexta* assembly (JHU_Msex_v1.0) that contains 70 Mb more genomic sequence and is 20-fold more continuous than the previous one. This allowed refining of gene models, identification and classification of repetitive DNA and transposable elements, enhancement of alignment of resequencing data and improved comparative genomics analysis. Our assembly consists of 470 Mb with a scaffold N50 of 14.2 Mb. We lifted over the original genome annotation to our new assembly and supplemented it with new high quality novel gene models including 794 previously un-annotated genes, including 47 novel serine proteases, constructed using publicly available RNA sequencing data. We demonstrate the utility of our new genome assembly through comparative genomics and gene expression analysis throughout *M. sexta* development cementing our assembly as a core resource for the lepidopteran community.

## Materials and methods

### Sample collection and sequencing

All DNA extraction and sample collection was done on a single male moth purchased as a pupa from Carolina Biological (Burlington, NC). On the day of emergence, the moth was sacrificed by placing it at 4°C for 10 minutes. The moth’s body, legs, head (including antennae and proboscis), and wings were snap-frozen in liquid nitrogen, then stored at –80°C prior to DNA extraction. High-quality genomic DNA was extracted from the legs and wings of the adult male moth using Qiagen G-tips. Briefly, we pulverized 14 mg of wing and leg tissue to a fine powder with a RETSCH CryoMill. DNA was purified from this powder using the Qiagen Genomic-tip 20/G kit with a modified lysis buffer consisting of 20 mM EDTA, 100 mM NaCl, 1% Triton-X, 33 mM Guanidine Thiocyanate, and 10 mM Tris-HCl. The 50°C lysis step was extended overnight. The quantity and quality of DNA were measured with Qubit 3.0 (Thermo Fisher Scientific, Inc., Carlsbad, CA, USA). We performed a total of four DNA extractions to generate all the Nanopore and Illumina data. Each extraction generated ∼2 μg of high-quality DNA which was used for library preparation and high throughput sequencing with Oxford Nanopore and Illumina platforms (Table 1).

**Table 1:** Sequencing summary statistics. A comparison of sequencing data collection for the JHU_Msex_V1.0 assembly versus the Msex_1.0 assembly

	Library type	Platform	Yield (Gb)	Library size (bp)	Number of reads
JHU_Msex_V1.0	Short read	Illumina Novaseq	32.83	2 × 150	219M
	Long read	ONT MinION	19.47	N50: 9,156	4.40M
	Hi-C	Illumina Novaseq	32.76	2 × 150	218M
Msex_1.0	Short read	454 Titanium fragment	14	fragment	26.5M
	Mate pair	454 mate pair	7	3,000	14.7M
	Mate pair	454 mate pair	2.5	8,000	6.3M
	BAC	Sanger	na	165,000	7,000

Oxford Nanopore sequencing libraries were prepared using the Ligation Sequencing 1 D Kit (Oxford Nanopore, Oxford, UK, SQK-LSK109) according to manufacturer's instructions and sequenced for 48 hours on three MinION R9.4.1 flow cells. Nanopore reads were base-called with Albacore Sequencing Pipeline Software (version 2.1.10). Sequencing runs from leg and wing tissues were run on separate flow cells and the data pooled for genome assembly purposes. To avoid any microbial contamination in the assembly, microbial read sequences identified by Centrifuge (v1.0.3-beta) were removed from Oxford Nanopore data (Kim *et al.* 2016). This resulted in a total of 4,404,206 reads yielding 19.5 Gb with a read length N50 of 9156 bp (Table 1; Supplementary Table S1 and Figure S1). For shotgun Illumina sequencing, a paired-end (PE) library was prepared with the Nextera DNA Flex Library Prep Kit from Illumina and sequenced on the Illumina NovaSeq6000 (Illumina, Inc., San Diego, CA, USA) yielding 219 M paired-end 150 (PE150) reads. A total of 32.83 Gb of Illumina data were generated and used for genome survey, correction, and evaluation. All sequencing data have been deposited at the NCBI SRA database under BioProject PRJNA658700.

### Hi-C

To assemble contigs into chromosome sized scaffolds, we generated Hi-C libraries using 200 mg of tissue from the head (including proboscis and antenna) of the same moth. The tissue was grounded with a mortar and pestle cooled by liquid nitrogen. We then followed the Phase Genomics Proximo HiC animal kit (Phase Genomics, Inc., Seattle, WA, USA) protocol. The quality of the resulting purified library was evaluated with Qubit 3.0 (Thermo Fisher Scientific, Inc.), and the Agilent 4200 TapeStation System (Agilent Technologies, Inc., Santa Clara, CA, USA). For a final QC step, we ran the library on a 2x100 cycle sequencing run on an Illumina MiSeq v2 (Illumina, Inc., San Diego, CA, USA). The qualified library was sequenced using the Illumina NovaSeq6000 (Illumina, Inc., San Diego, CA, USA) again generating 150 bp paired-end reads. A total of 218 M reads (32.76 Gb) were generated on the NovaSeq6000 and used for the subsequent Hi-C analysis (NCBI SRA BioProject PRJNA658700).

### Genome assembly and polishing

**Figure 1:**
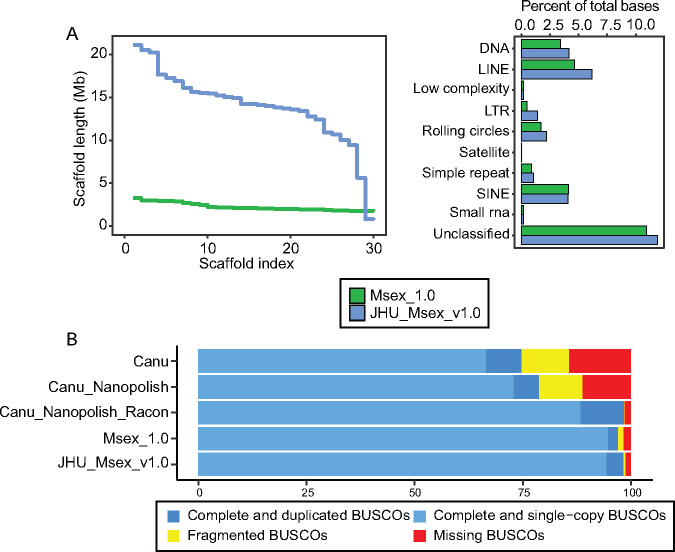
Final assembly metrics. (A) (Left) NGx plot for final JHU_Msex_v1.0 assembly after scaffolding compared to the old Msex_1.0 assembly. Plot represents the largest 30 scaffolds from each assembly. (Right) Repeat annotation comparisons JHU_Msex_v1.0 compared to MSex_1.0. (B) BUSCO results from insecta odbv10 database. Comparing the raw Canu assembly, Nanopolished Canu assembly, Racon and Nanopolished Canu assembly, the final scaffolded and polished assembly (JHU_Msex_v1.0) and the Msex_1.0 assembly.

Nanopore sequencing reads were used to construct an initial assembly using Canu (v2.0) (Koren *et al.* 2017). This initial assembly contained 5381 contigs with a contig N50 of 424,554bp. A contig is a continuous stretch of DNA sequence and a scaffold is the connection of multiple contigs filled in with gaps. Longer contigs and scaffolds indicate more continuous genome assemblies. Next, we polished the Canu assembly by first aligning all nanopore data to the draft assembly with Minimap2 (2.17-r943-dirty) and running a single iteration of the nanopolish (v0.11.1) consensus module (Loman *et al.* 2015; Li 2018). Nanopolish uses a hidden Markov model (HMM) to examine the raw electrical data for possible improvements to the consensus sequence. The polished nanopore draft assembly was then error-corrected with 32.38 Gb of shotgun Illumina data using Bowtie2 (v2.4.1) for alignment and Racon (v1.3.3) for polishing (Langmead and Salzberg 2012; Vaser *et al.* 2017). We iteratively polished the Canu assembly with Racon, and after each iteration, the polished genome was aligned to the previous genome and SNPs were called with Mummer4 to evaluate the number of bases changed (Marçais *et al.* 2018). To assess the improvements made by polishing, we ran BUSCO, a quantitative assessment of genome assembly and completeness based on evolutionarily informed expectations of gene content (Simão *et al.* 2015). Errors in the assembly cause genes to go undetected by BUSCO, therefore being labeled as “missing” or “fragmented” BUSCOs. A single iteration of nanopolish improved BUSCO completeness scores by 4.0% (Figure 1B). A single iteration of Racon improved BUSCO completeness scores by 19.6% (Figure 1B) and further iterations had minimal improvements (Supplementary Figure S3).

### Chromosome assembly

To improve alignment of Hi-C libraries, we trimmed 150 bp reads to 75 bp with TrimGalore (v0.6.0) (–hardtrim5 75) (http://www.bioinformatics.babraham.ac.uk/projects/trim_galore/). To connect the polished contigs into chromosome-scale scaffolds, we used the Hi-C data with the 3 D-DNA (v180922) and Juicer (v1.6) pipelines (–mode diploid –editor-repeat-coverage 3) (Dudchenko *et al.* 2017). 3 D-DNA generated 4057 scaffolds of which 28 contain 86% (404.70 Mb) of the total input sequence length, consistent with 28 chromosomes seen on the previous karyotype analyses (Supplementary Figure S2) (Yasukochi *et al.* 2009). The final chromosome level assembly of *M. sexta* is 470 Mb (467 Mb without gaps) with a contig N50 of 402 Kb and a scaffold N50 of 14.2 Mb, a considerable improvement when compared to Msex_1.0 (Table 2, Figure 1A). We evaluated the final assembly for completeness with BUSCO insecta_odb10 (Figure 1B;Supplementary Table S2). We note that our final scaffolded *M. sexta* assembly is highly complete and contiguous, containing 98.1% complete insecta BUSCOs (Figure 1B).

**Table 2:** Final assembly statistics

Statistic	JHU_Msex_V1.0	Msex_1.0
Total contig length	468,966,500	399,658,702
Number of contigs	6,517	38,553
Contig N50	402,416	40,289
Largest contig	2,620,325	401,033
Total scaffold length	470,051,811	419,427,777
Number of scaffolds	4,057	20,869
Scaffold N50	14,248,853	664,006
Largest scaffold	21,114,789	3,253,989

A comparison of assembly continuity metrics between the JHU_Msex_V1.0 assembly and the Msex_1.0 assembly. Contig and scaffold N50 is a weighted median statistic such that 50% of the entire assembly is contained in contigs or scaffolds equal to or larger than this value, therefore larger N50 values indicate more continuous assemblies. We note a contig N50 that is 20-fold greater, and the largest scaffold 7 times larger in the JHU_Msex_V1.0 compared to Msex_1.0.

### Repetitive elements

With the genome in hand, next we examined the repetitive elements in the *M. sexta* genome assembly. We used RepeatModeler2 to generate *de novo* repeat libraries (Flynn *et al*. 2020). Next, we performed homology based repeat masking using the *de novo* libraries in combination with the curated Metazoan library with RepeatMasker. We detected 159 Mb of repetitive sequence representing 33.91% of the genome, which is greater than the 28.84% detected from the Msex_1.0 assembly (Figure 1A, Supplementary Table S3). This proportion of repetitive sequence is considerably smaller than that of the domestic silkworm genome (*Bombyx mori)* (∼46.84% of 460 Mb genome) (Kawamoto *et al.* 2019), but notably greater than reported for the monarch butterfly, *D. plexippus* (∼13% of 273 Mb) (Zhan *et al.* 2011). Among classified repeats, LINE elements were the most abundant superfamily found in the *M. sexta* genome, with the L2 LINE element being the most common (Supplementary Table S4). However, the overwhelming majority of masked regions dispersed throughout the genome corresponded to complex repetitive sequences yet to be characterized (11.88%), which is consistent with the domestic silkworm (11.73%).

### RNA-seq data

Sixty-seven RNA-seq data sets from *M. sexta* were downloaded from the sequence read archive (SRA) with accession numbers listed in Supplementary Data S1 (Cao and Jiang 2017). All RNA-seq sequences were first trimmed with TRIMMOMATIC (SLIDINGWINDOW:4:20 LEADING:10 TRAILING:10 MINLEN:50) prior to alignment to the JHU_Msex_V1.0 assembly with HISAT2 using default parameters (Bolger *et al.* 2014; Kim *et al.* 2019). An average of 96.2% of reads aligned from all libraries (Supplementary Data S1). These bam files were used for both gene annotation and expression analyses.

### Gene annotation

Gene annotation was primarily accomplished via a liftover of the Msex_1.0 annotations from the NCBI annotation (GCA_000262585.1) with Liftoff, a tool that maps annotations between closely related species or, in this case, two assemblies of the same species (Shumate and Salzberg 2020). Liftoff successfully mapped 39,957 of the 41,110 original features to our new *M. sexta* assembly. We supplemented these annotations with gene models assembled from RNA-seq data and *ab initio* gene predictions made by AUGUSTUS (Stanke *et al.* 2006a; Cao and Jiang 2017) Aligned RNA-seq data was input into the BRAKER pipeline which relies on GenMark-ES/ET and AUGUSTUS to generate gene predictions (Stanke *et al.* 2006b, 2008; Li *et al.* 2009; Barnett *et al.* 2011; Gremme *et al.* 2013; Buchfink *et al.* 2015; Hoff *et al.* 2016, 2019). Additionally, we assembled the full transcriptome from the RNA-seq data with Stringtie2 (Kovaka *et al.* 2019). Both the *de novo* transcriptome assembly and *ab initio* gene predictions are prone to assembling erroneous transcripts. In order to only filter for high quality transcripts, we first aligned both the Stringtie2 and AUGUSTUS gene models to the Liftoff gene models with GFFCompare (Pertea and Pertea 2020). To identify unannotated genes, we examined transcripts supported by evidence from both the Stringtie2 assembly and the AUGUSTUS prediction, but not contained in the Liftoff annotation. We identified 794 such transcripts to add to the annotation. Overall, we identified 25,256 total genes compared to the 24,462 contained in the NCBI annotation of Msex_1.0.

### Data availability

The JHU_Msex_v1.0 genome, corresponding annotation, Supplementary Data S1 and S2, and Supplementary material is available on Zenodo (https://doi.org/10.5281/zenodo.4302295). This Whole Genome Shotgun project has been deposited at DDBJ/ENA/GenBank under the accession JACVES000000000. The version described in this paper is version JACVES010000000. Whole genome sequencing data are available in the NCBI SRA database under BioProject PRJNA658700. The scripts we used in this article, including the genome assembly, genome polishing, repeat annotation, and genome assessments, are available on Github (https://github.com/timplab/moth).

## Results

### Identification of orthologous genes and phylogenetic tree construction

To identify candidate coding regions and generate predicted protein sequences, we ran TransDecoder (https://github.com/TransDecoder/TransDecoder/wiki) on our annotation. The generated protein sequences were used for determining phylogenetic relationships between *M. sexta* and other lepidopteran species by measuring pairwise sequence similarity. We used OrthoFinder (v2.3.12) to identify orthologous gene clusters in *M. sexta* and five other related lepidopteran species: *Bombyx mori* (domestic silkworm, GCF_000151625.1), *Plutella xylostella* (diamondback moth, GCF_000330985.1), *Papilio polytes* (common Mormon, GCF_000836215.1), *Papilio xuthus* (Asian swallowtail, GCF_000836235.1), and *Danaus plexippus plexippus* (monarch butterfly, GCF_009731565.1) (Bertone *et al.* 1991; Van Dongen 2000; Price *et al.* 2010; Katoh and Standley 2013; Kelly and Maini 2013; Buchfink *et al.* 2015; Emms and Kelly 2015, 2017, 2018, 2019; Lefort *et al.* 2015; Huerta-Cepas *et al.* 2016). OrthoFinder groups genes into orthogroups, sets of genes descended from a single gene in the species, the last common ancestor based on their sequence similarity.

**Figure 2:**
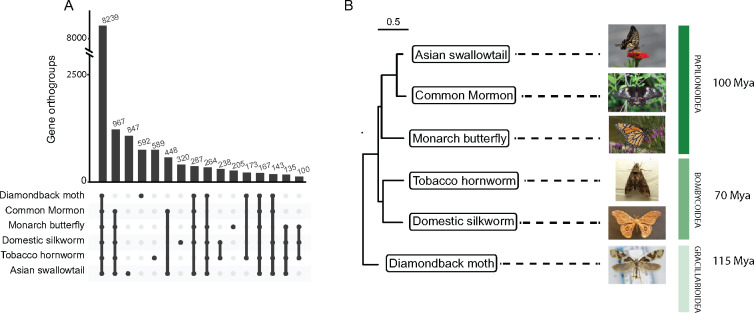
Phylogenetic relationships. (A) Upset plot illustrating the number of shared orthogroups between the six species. Bars with less than 100 orthogroups were removed. (B) Phylogenetic tree generated from orthogroup comparisons. Age of divergence in Mya (million years ago) was collected from Kawahara *et al.* (2019).

OrthoFinder identified 15,428 orthogroups containing 120,091 total genes. Of these, 8239 (53.4%) were shared between all six species and 1783 were shared and single copy (Figure 2A;Supplementary Figure S4). Furthermore, the tobacco hornworm had the most shared orthogroups with the domestic silkworm (Figure 2A). The phylogenetic tree indicated the tobacco hornworm is more closely related to the domestic silkworm (Figure 2B). This closer relationship is expected due to the fact that both are members of the Lepidopteran superfamily Bombycoidea, however Bombycidae (domestic silkworm family) and Sphingidae (tobacco hornworm family) diverged about 50–60 Mya (Kitching *et al.* 2018; Kawahara *et al.* 2019).

### Expression analysis

**Figure 3:**
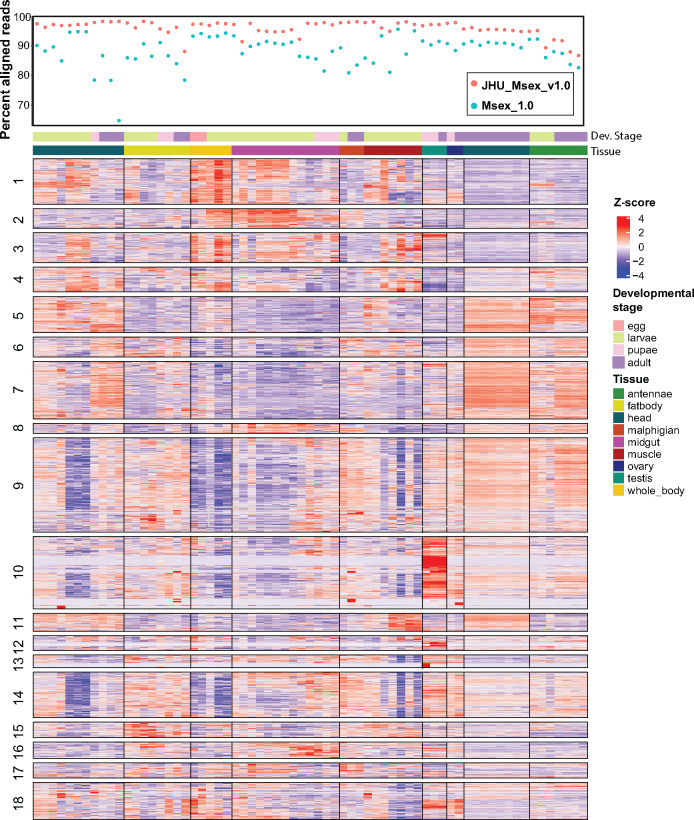
Gene expression clustering Top panel is the percent aligned from each RNA-seq library to either our JHU_Msex_v1.0 or Msex_1.0. Expression matrix is the Z-score of the rlog expression transformation for all highly expressed genes. Genes were clustered by Z-score into 18 clusters by euclidean distance clustering. Libraries are in order shown in Supplementary Data S1. Statistically significant enriched GO terms for each cluster are listed in Supplementary Data S2. Results can also be shown as gene expression matrix with libraries ordered by increasing developmental stage (Supplementary Figure S6)

Using the same publicly available RNA-seq data (Supplementary Data S1), we wanted to examine how our assembly improved results of gene expression analysis. The average alignment rate from each dataset to JHU_Msex_v1.0 was 96.16% and to the Msex_1.0 assembly was 88.23% (Figure 3; Supplementary Data S1). We noted that paired-end data sets aligned significantly better to our assembly than to the old assembly with an increase of 11.92% of reads aligned as compared to single-end libraries where we only saw an improvement 4.06% of reads aligning. Gene abundance values were calculated using our annotation with the Stringtie2 gene abundance pipeline and the regularized log transformation (rlog) from DEseq2 (Love *et al.* 2014). The rlog transformation takes the read count data from Stringtie2 and accounts for differences in sequencing depth, RNA composition, heteroskedasticity, and large dynamic range (Love *et al.* 2014). To filter out genes with low expression in all tissues, we only retained genes if they had an rlog score of greater than three in at least one library. We then calculated the z-score of the rlog values and computed a Euclidean distance matrix to perform hierarchical clustering, which resulted in 18 gene clusters (Figure 3). To annotate the gene clusters, we ran Interproscan5 to add GO annotations for all genes then used TopGO for GO term enrichment calculations within each gene cluster (Alexa *et al.* 2006; Jones *et al.* 2014). Significantly enriched GO terms for biological process (BP), molecular function (MF), and cellular compartment (CC) were calculated by the Fisher’s exact test and determined significant if the *P*-value was less than 0.05 (Supplementary Data S2). We note that these RNA-seq datasets were collected from different insects without biological replicates and throughout multiple RNA-seq studies and while results show interesting patterns, we cannot make definitive biological conclusions.

As expected, the enriched GO terms were well correlated with the expression pattern of gene clusters. For example, cluster 10 has the highest expression in the ovaries and testes and the most significantly enriched terms of cluster 10 include microtubule-based process, lncRNA processing and transcription (Supplementary Data S2). Sperm generation and meiosis requires continuous cell division and chromosome segregation, aided by the movement of microtubules. Additionally, the flagellar motor of sperm cells that aids in their motility is made up of microtubules. Cluster 7 includes genes, which are highly expressed in the antennae and in the adult head. Consistent with these tissues being responsible for sensory perception, GO annotations are enriched for GTPase activity and odorant binding (Supplementary Data S2). Insects have a repertoire of sensory driven behaviors and odorant receptor (ORs) genes code for an entire family of G protein coupled receptors (GPCRs), a class of transmembrane proteins that have GTPase activity (Benton 2006).

We also noted that both clusters 2 and 16 were highly expressed in the midgut tissue, albeit, at different developmental stages. Cluster 2 contains genes involved in proteolysis, metabolic processes, and catalytic activity, and the expression is high in the larval stages of the midgut until larval stage five (L5), the pre-wandering stage. In the stages after L5, expression of cluster 16 genes increases in the midgut (Supplementary Data S2). Cluster 16 contains genes involved in the negative regulation of metabolic processes and apoptotic processes (Supplementary Data S2). During the process of metamorphosis the moth larvae will stop feeding in the pre-wandering stage, which coincides with the decrease in the expression of the cluster 2 genes and increase in the negative regulation of metabolic processes. During the pupation process, the insects experience massive tissue reorganization including death of major organs (Zakeri *et al.* 1996; Dai and Gilbert 1997). This process is likely mediated by an apoptotic or an autophagic genes, eg., autophagy-related protein 8 (ATG8), as seen with *B. mori* (Romanelli *et al.* 2016).

### Metamorphosis in the midgut

**Figure 4:**
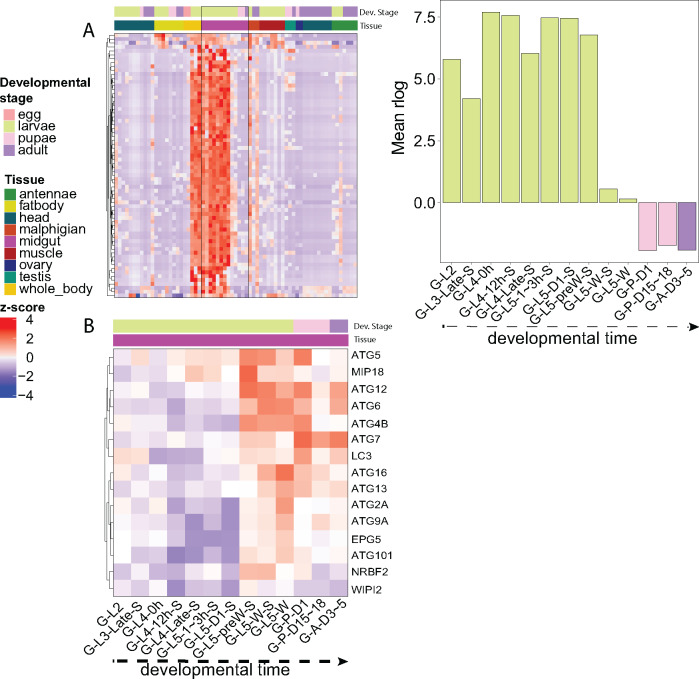
Gene expression in the midgut. (A) (Left) Heatmap of Z-scores for expression of digestive proteases. (Right) Quantification of midgut digestive protease expression throughout development. Library naming nomenclature was derived from Cao and Jiang (2017). The first part of the library names indicates that the libraries are made from midgut (G). The second part indicates major stages of the insect, *i.e.*, embryo (E), 1st to 5th instar larvae (L1 − L5), pupae (P), and adults (A). In the third part, “D” stands for day, “h” for hour, “preW” for pre-wandering, “W” for wandering. “S” in the last part of library names indicates single-end sequencing; no “S” in the end indicates paired-end sequencing. The libraries present as follows: midgut (G) (2nd L; 3rd L; 4th L, 0 h; 4th L, 12 h; 4th L, late; 5th L, 1–3 h; 5th L, 24 h;. 5th L, preW; 5th L, W; P, D1; P, D15–18; A, D3–5). (B) (Left) Heatmap of expression Z-score of midgut autophagic genes throughout development. Gene names were assigned based on the NCBI GCA_000262585.1. Genes not present in this annotation were functionally annotated with Interproscan5 and assigned gene names. All genes in the heatmap were annotated as autophagy Gene Ontology Term (GO:0006914)

To showcase our new reference genome, we focused on gene expression changes occurring in the midgut during *M. sexta* development. We focused on serine proteases, which are proteins involved in catalytic cleavage of other proteins, significant in physiological processes like digestion, development, and defense. We ran Interproscan5 to identify putative serine proteases by Pfam classification (Finn *et al.* 2014). We identified 240 proteins with potential serine protease domains (PF00089), compared to the 193 identified in Msex_1.0 (Kanost *et al.* 2016). Of the serine proteases we identified 76 that were highly expressed primarily in the midgut, compared to the 68 gut proteases previously identified in Msex_1.0 (Kanost *et al.* 2016) (Figure 4A). Upon the cessation of feeding in the L5 pre-wandering stage, expression of the digestive proteases declines rapidly as the insects prepare for pupation (Figure 4, A and B). With our new highly contiguous genome assembly, we were able to annotate large protease gene arrays on scaffolds 13 (60 kb) and 18 (1 Mb).

To further investigate the mechanism of midgut tissue reorganization during metamorphosis, we analyzed the gene expression patterns of genes involved in both apoptosis and autophagy in the *M. sexta* midgut throughout development. We generated lists of autophagy and apoptosis related genes using the GO annotations GO0006914 and GO0006915 from Interproscan5 as those encompassed multiple Pfam domains and gave a more complete list of genes related to these pathways. We did not note a distinct expression pattern of apoptotic genes throughout midgut development (Supplementary Figure S5). However, we did identify an increase in expression of genes within the autophagic pathway beginning at the L5 pre-wandering stage (G-L5-preW-S), which coincides with the decline in digestive protease expression at the L5 wandering stage (G-L5-W-S/G-L5-W) (Figure 4B). These results indicate the potential involvement of the autophagic pathway in *M. sexta* midgut during metamorphosis, at the same time in development that autophagy has been shown to remodel the midgut of other insects like fruit flies (Denton *et al.* 2009), silkmoths (Romanelli *et al.* 2016), and sand flies (Malta *et al.* 2017).

## Discussion

Our results have leveraged short and long read sequencing technology to assemble a highly contiguous reference genome of *M. sexta*. We lifted over the original genome annotations to this improved assembly, maintaining functional annotations and gene IDs for researchers currently studying *M. sexta* genes. We believe the improved assembly will aid current and future studies using *M. sexta* as a model system for research on fundamental processes in insect physiology and biochemistry. We note that this assembly is a male (ZZ) system and does not contain sequence for the female W chromosome. Future work could assembly the female W chromosome, as well as develop a deeper understanding of diversity in the species population.

## References

[jkaa047-B1] Alexa A , Rahnenführer J, Lengauer T. 2006. Improved scoring of functional groups from gene expression data by decorrelating GO graph structure. Bioinformatics. 22:1600–1607. 16606683 10.1093/bioinformatics/btl140

[jkaa047-B2] Barnett DW , Garrison EK, Quinlan AR. 2011. BamTools: a C++ API and toolkit for analyzing and managing BAM files. 10.1093/bioinformatics/btr174 PMC3106182 21493652

[jkaa047-B311] Bioinformatics 27: 1691–1692. 10.1093/bioinformatics/btr174 PMC3106182 21493652

[jkaa047-B3] Benton R. 2006. On the origin of smell: odorant receptors in insects. Cell Mol Life Sci. 63:1579–1585. 16786219 10.1007/s00018-006-6130-7 PMC11136275

[jkaa047-B5] Bertone P , Stolc V, Royce TE, Rozowsky JS, Urban AE, et al 1991. Basic local alignment search tool. J Mol Biol. 215:403–410. 10.1016/S0022-2836(05)80360-2 2231712

[jkaa047-B6] Boer G , Hanson FE. 1987. Feeding responses to solanaceous allelochemicals by larvae of the tobacco hornworm, *Manduca sexta*. Entomol Exp Appl. 45:123–131.

[jkaa047-B7] Bolger AM , Lohse M, Usadel B. 2014. Trimmomatic: a flexible trimmer for Illumina sequence data. Bioinformatics. 30:2114–2120. 24695404 10.1093/bioinformatics/btu170 PMC4103590

[jkaa047-B8] Buchfink B , Xie C, Huson DH. 2015. Fast and sensitive protein alignment using DIAMOND. Nat Methods. 12:59–60. 25402007 10.1038/nmeth.3176

[jkaa047-B9] Cao X , He Y, Hu Y, Zhang X, Wang Y, et al 2015. Sequence conservation, phylogenetic relationships, and expression profiles of nondigestive serine proteases and serine protease homologs in *Manduca sexta*. Insect Biochem Mol Biol. 62:51–63. 25530503 10.1016/j.ibmb.2014.10.006 PMC4474797

[jkaa047-B10] Cao X , Jiang H. 2017. An analysis of 67 RNA-seq datasets from various tissues at different stages of a model insect, *Manduca sexta*. BMC Genomics. 18:796. 29041902 10.1186/s12864-017-4147-y PMC5645894

[jkaa047-B11] Dai JD , Gilbert LI. 1997. Programmed cell death of the prothoracic glands of *Manduca sexta* during pupal–adult metamorphosis. Insect Biochem Mol Biol. 27:69–78. 9061930 10.1016/s0965-1748(96)00068-9

[jkaa047-B12] Denton D , Shravage B, Simin R, Mills K, Berry DL, et al 2009. Autophagy, not apoptosis, is essential for midgut cell death in *Drosophila*. Curr Biol. 19:1741–1746. 19818615 10.1016/j.cub.2009.08.042 PMC2783269

[jkaa047-B13] Denton JF , Lugo-Martinez J, Tucker AE, Schrider DR, Warren WC, et al 2014. Extensive error in the number of genes inferred from draft genome assemblies. PLoS Comput Biol. 10:e1003998. 25474019 10.1371/journal.pcbi.1003998 PMC4256071

[jkaa047-B14] Dudchenko O , Batra SS, Omer AD, Nyquist SK, Hoeger M, et al 2017. *De novo* assembly of the *Aedes aegypti* genome using Hi-C yields chromosome-length scaffolds. Science. 356:92–95. 28336562 10.1126/science.aal3327 PMC5635820

[jkaa047-B15] Emms DM , Kelly S. 2015. OrthoFinder: solving fundamental biases in whole genome comparisons dramatically improves orthogroup inference accuracy. Genome Biol. 16:157. 26243257 10.1186/s13059-015-0721-2 PMC4531804

[jkaa047-B16] Emms DM , Kelly S. 2017. STRIDE: species tree root inference from gene duplication events. Mol Biol Evol. 34:3267–3278. 29029342 10.1093/molbev/msx259 PMC5850722

[jkaa047-B17] Emms DM , Kelly S. 2018. STAG: Species Tree Inference from All Genes. bioRxiv. doi: 10.1101/267914 (Preprint posted Feb 19,2018)

[jkaa047-B18] Emms DM , Kelly S. 2019. OrthoFinder: phylogenetic orthology inference for comparative genomics. Genome Biol. 20:238. 31727128 10.1186/s13059-019-1832-y PMC6857279

[jkaa047-B19] Finn RD , Bateman A, Clements J, Coggill P, Eberhardt RY, et al 2014. Pfam: the protein families database. Nucl Acids Res. 42:D222–D230. 24288371 10.1093/nar/gkt1223 PMC3965110

[jkaa047-B20] Flynn JM , Hubley R, Goubert C, Rosen J, Clark AG, et al 2020. RepeatModeler2: automated genomic discovery of transposable element families. Proc Natl Acad Sci USA. 117:19451–9457 10.1073/pnas.1921046117 PMC7196820 32300014

[jkaa047-B21] Gremme G , Steinbiss S, Kurtz S. 2013. GenomeTools: a comprehensive software library for efficient processing of structured genome annotations. IEEE/ACM Trans Comput Biol and Bioinf. 10:645–656. 10.1109/TCBB.2013.68 24091398

[jkaa047-B22] Gu L , Reilly PF, Lewis JJ, Reed RD, Andolfatto P, et al 2019. Dichotomy of dosage compensation along the Neo Z chromosome of the monarch butterfly. Curr Biol. 29(e3):4071–4077. 31735674 10.1016/j.cub.2019.09.056 PMC6901105

[jkaa047-B23] Hoff KJ , Lange S, Lomsadze A, Borodovsky M, Stanke M. 2016. BRAKER1: unsupervised RNA-seq-based genome annotation with GeneMark-ET and AUGUSTUS: table 1. Bioinformatics. 32:767–769. 26559507 10.1093/bioinformatics/btv661 PMC6078167

[jkaa047-B24] Hoff KJ , Lomsadze A, Borodovsky M, Stanke M. 2019. Whole-genome annotation with BRAKER. Methods Mol Biol. 1962: 65–95. 31020555 10.1007/978-1-4939-9173-0_5 PMC6635606

[jkaa047-B25] Huerta-Cepas J , Serra F, Bork P. 2016. ETE 3: reconstruction, analysis, and visualization of phylogenomic data. Mol Biol Evol. 33:1635–1638. 26921390 10.1093/molbev/msw046 PMC4868116

[jkaa047-B26] Jiang H , Vilcinskas A, Kanost MR. 2010. Immunity in lepidopteran insects. Adv Exp Med Biol. 708:181–204. 21528699 10.1007/978-1-4419-8059-5_10 PMC9284565

[jkaa047-B27] Jones MEE , Haire MF, Kloetzel P-M, Mykles DL, Schwartz LM. 1995. Changes in the structure and function of the multicatalytic proteinase (proteasome) during programmed cell death in the intersegmental muscles of the hawkmoth, *Manduca sexta*. Dev Biol. 169:436–447. 7781889 10.1006/dbio.1995.1159

[jkaa047-B28] Jones P , Binns D, Chang H-Y, Fraser M, Li W, et al 2014. InterProScan 5: genome-scale protein function classification. Bioinformatics. 30:1236–1240. 24451626 10.1093/bioinformatics/btu031 PMC3998142

[jkaa047-B29] Kanost MR , Arrese EL, Cao X, Chen Y-R, Chellapilla S, et al 2016. Multifaceted biological insights from a draft genome sequence of the tobacco hornworm moth, *Manduca sexta*. Insect Biochem Mol Biol. 76:118–147. 27522922 10.1016/j.ibmb.2016.07.005 PMC5010457

[jkaa047-B30] Kanost MR , Jiang H, Yu X-Q. 2004. Innate immune responses of a lepidopteran insect, *Manduca sexta*. Immunol Rev. 198:97–105. 15199957 10.1111/j.0105-2896.2004.0121.x

[jkaa047-B31] Katoh K , Standley DM. 2013. MAFFT multiple sequence alignment software version 7: improvements in performance and usability. Mol Biol Evol. 30:772–780. 23329690 10.1093/molbev/mst010 PMC3603318

[jkaa047-B32] Kawahara AY , Plotkin D, Espeland M, Meusemann K, Toussaint EFA, et al 2019. Phylogenomics reveals the evolutionary timing and pattern of butterflies and moths. Proc Natl Acad Sci USA. 116:22657–22663. 31636187 10.1073/pnas.1907847116 PMC6842621

[jkaa047-B33] Kawamoto M , Jouraku A, Toyoda A, Yokoi K, Minakuchi Y, et al 2019. High-quality genome assembly of the silkworm, *Bombyx mori*. Insect Biochem Mol Biol. 107:53–62. 30802494 10.1016/j.ibmb.2019.02.002

[jkaa047-B34] Kay I , Patel M, Coast GM, Totty NF, Mallet AI, et al 1992. Isolation, characterization and biological activity of a CRF-related diuretic peptide from *Periplaneta americana* L. Regul Pept. 42:111–122. 1337794 10.1016/0167-0115(92)90091-8

[jkaa047-B35] Kelly S , Maini PK. 2013. DendroBLAST: approximate phylogenetic trees in the absence of multiple sequence alignments. PLoS One. 8:e58537. 23554899 10.1371/journal.pone.0058537 PMC3598851

[jkaa047-B36] Kim D , Paggi JM, Park C, Bennett C, Salzberg SL. 2019. Graph-based genome alignment and genotyping with HISAT2 and HISAT-genotype. Nat Biotechnol. 37:907–915. 31375807 10.1038/s41587-019-0201-4 PMC7605509

[jkaa047-B37] Kim D , Song L, Breitwieser FP, Salzberg SL. 2016. Centrifuge: rapid and sensitive classification of metagenomic sequences. Genome Res. 26:1721–1729. 27852649 10.1101/gr.210641.116 PMC5131823

[jkaa047-B38] Kitching IJ , Rougerie R, Zwick A, Hamilton CA, St Laurent RA, et al 2018. A global checklist of the Bombycoidea (Insecta: Lepidoptera). Biodivers Data J. 6:e22236. 10.3897/BDJ.6.e22236 PMC5904559 29674935

[jkaa047-B39] Koren S , Walenz BP, Berlin K, Miller JR, Bergman NH, et al 2017. Canu: scalable and accurate long-read assembly via adaptive k-mer weighting and repeat separation. Genome Res. 27:722–736. 28298431 10.1101/gr.215087.116 PMC5411767

[jkaa047-B40] Kovaka S , Zimin AV, Pertea GM, Razaghi R, Salzberg SL, et al 2019. Transcriptome assembly from long-read RNA-seq alignments with StringTie2. Genome Biol. 20:278. 31842956 10.1186/s13059-019-1910-1 PMC6912988

[jkaa047-B41] Langmead B , Salzberg SL. 2012. Fast gapped-read alignment with Bowtie 2. Nat Methods. 9:357–359. 22388286 10.1038/nmeth.1923 PMC3322381

[jkaa047-B42] Lefort V , Desper R, Gascuel O. 2015. FastME 2.0: a comprehensive, accurate, and fast distance-based phylogeny inference program: table 1. Mol Biol Evol. 32:2798–2800. 26130081 10.1093/molbev/msv150 PMC4576710

[jkaa047-B44] Li H. 2018. Minimap2: pairwise alignment for nucleotide sequences. Bioinformatics. 34:3094–3100. 29750242 10.1093/bioinformatics/bty191 PMC6137996

[jkaa047-B45] Li H , Handsaker B, Wysoker A, Fennell T, Ruan J, 1000 Genome Project Data Processing Subgroup, et al 2009. The sequence alignment/map format and SAMtools. Bioinformatics. 25:2078–2079. 19505943 10.1093/bioinformatics/btp352 PMC2723002

[jkaa047-B46] Lockshin RA , Williams CM. 1965. Programmed cell death—V. Cytolytic enzymes in relation to the breakdown of the intersegmental muscles of silkmoths. J Insect Physiol. 11:831–844. 5828992 10.1016/0022-1910(65)90186-1

[jkaa047-B47] Loman NJ , Quick J, Simpson JT. 2015. A complete bacterial genome assembled *de novo* using only nanopore sequencing data. Nat Methods. 12:733–735. 26076426 10.1038/nmeth.3444

[jkaa047-B48] Love MI , Huber W, Anders S. 2014. Moderated estimation of fold change and dispersion for RNA-seq data with DESeq2. Genome Biol. 15:550. 25516281 10.1186/s13059-014-0550-8 PMC4302049

[jkaa047-B49] Lyons N , Softley I, Balfour A, Williamson C, O’Brien HE, et al 2020. Tobacco hornworm (*Manduca sexta*) caterpillars as a novel host model for the study of fungal virulence and drug efficacy. Virulence 11: 1075–1089. 10.1080/21505594.2020.1806665 PMC7549948 32842847

[jkaa047-B50] Malta J , Heerman M, Weng JL, Fernandes KM, Martins GF, et al 2017. Midgut morphological changes and autophagy during metamorphosis in sand flies. Cell Tissue Res. 368:513–529. 28285352 10.1007/s00441-017-2586-z

[jkaa047-B51] Marçais G , Delcher AL, Phillippy AM, Coston R, Salzberg SL, et al 2018. MUMmer4: a fast and versatile genome alignment system. PLoS Comput Biol. 14:e1005944. 29373581 10.1371/journal.pcbi.1005944 PMC5802927

[jkaa047-B52] Mechaber WL , Capaldo CT, Hildebrand JG. 2002. Behavioral responses of adult female tobacco hornworms, *Manduca sexta*, to hostplant volatiles change with age and mating status. J Insect Sci. 2:1–8. 15455039 PMC355905

[jkaa047-B53] Pertea G , Pertea M. 2020. GFF Utilities: GffRead and GffCompare. F1000Res. 9:304. 10.12688/f1000research.23297.1 PMC7222033 32489650

[jkaa047-B54] Price MN , Dehal PS, Arkin AP. 2010. FastTree 2–approximately maximum-likelihood trees for large alignments. PLoS One. 5:e9490. 20224823 10.1371/journal.pone.0009490 PMC2835736

[jkaa047-B55] Romanelli D , Casartelli M, Cappellozza S, de Eguileor M, Tettamanti G. 2016. Roles and regulation of autophagy and apoptosis in the remodelling of the lepidopteran midgut epithelium during metamorphosis. Sci. Rep. 6:32939–32946 27609527 10.1038/srep32939 PMC5016986

[jkaa047-B56] Shumate A , Salzberg SL. 2020. Liftoff: an accurate gene annotation mapping tool. bioRxiv 2020.06.24.169680.

[jkaa047-B57] Simão FA , Waterhouse RM, Ioannidis P, Kriventseva EV, Zdobnov EM. 2015. BUSCO: assessing genome assembly and annotation completeness with single-copy orthologs. Bioinformatics. 31:3210–3212. 26059717 10.1093/bioinformatics/btv351

[jkaa047-B58] Stanke M , Diekhans M, Baertsch R, Haussler D. 2008. Using native and syntenically mapped cDNA alignments to improve *de novo* gene finding. Bioinformatics. 24:637–644. 18218656 10.1093/bioinformatics/btn013

[jkaa047-B59] Stanke M , Keller O, Gunduz I, Hayes A, Waack S, et al 2006a. AUGUSTUS: ab initio prediction of alternative transcripts. Nucleic Acids Res. 34:W435–W439. 16845043 10.1093/nar/gkl200 PMC1538822

[jkaa047-B60] Stanke M , Schöffmann O, Morgenstern B, Waack S. 2006b. Gene prediction in eukaryotes with a generalized hidden Markov model that uses hints from external sources. BMC Bioinformatics. 7:62. 16469098 10.1186/1471-2105-7-62 PMC1409804

[jkaa047-B61] Truman JW , Reiss SE. 1995. Neuromuscular metamorphosis in the moth *Manduca sexta*: hormonal regulation of synapses loss and remodeling. J Neurosci. 15:4815–4826. 7623113 10.1523/JNEUROSCI.15-07-04815.1995 PMC6577907

[jkaa047-B62] Van Dongen S. 2000. Graph clustering by flow simulation [Ph.D. thesis]. Utrecht, The Netherlands: University of Utrecht.

[jkaa047-B63] Vaser R , Sović I, Nagarajan N, Šikić M. 2017. Fast and accurate *de novo* genome assembly from long uncorrected reads. Genome Res. 27:737–746. 28100585 10.1101/gr.214270.116 PMC5411768

[jkaa047-B64] Wieczorek H , Grüber G, Harvey WR, Huss M, Merzendorfer H. 1999. The plasma membrane H+-V-ATPase from tobacco hornworm midgut. J Bioenerg Biomembr. 31:67–74. 10340850 10.1023/a:1005448614450

[jkaa047-B65] Yasukochi Y , Tanaka-Okuyama M, Shibata F, Yoshido A, Marec F, et al 2009. Extensive conserved synteny of genes between the karyotypes of *Manduca sexta* and *Bombyx mori* revealed by BAC-FISH mapping. PLoS One. 4:e7465. 19829706 10.1371/journal.pone.0007465 PMC2759293

[jkaa047-B66] Zakeri Z , Quaglino D, Latham T, Woo K, Lockshin RA. 1996. Programmed cell death in the tobacco hornworm, *Manduca sexta*: alteration in protein synthesis. Microsc Res Tech. 34:192–201. 8743407 10.1002/(SICI)1097-0029(19960615)34:3<192::AID-JEMT2>3.0.CO;2-S

[jkaa047-B67] Zhan S , Merlin C, Boore JL, Reppert SM. 2011. The monarch butterfly genome yields insights into long-distance migration. Cell. 147:1171–1185. 22118469 10.1016/j.cell.2011.09.052 PMC3225893

